# An Innovative Linear Wireless Sensor Network Reliability Evaluation Algorithm

**DOI:** 10.3390/s25010285

**Published:** 2025-01-06

**Authors:** Tao Ma, Huidong Guo, Xin Li

**Affiliations:** 1 College of Information Science and Engineering, Shenyang University of Technology, Shenyang 110167, China; matao@smail.sut.edu.cn (T.M.); lixin@sut.edu.cn (X.L.); 2 Shenyang Key Laboratory of Advanced Computing and Application Innovation, Shenyang 110167, China

**Keywords:** LWSNs, reliability evaluation, performance level, failure probability, number splitting

## Abstract

In recent years, wireless sensor networks (WSNs) have become a crucial technology for infrastructure monitoring. To ensure the reliability of monitoring services, evaluating the network’s reliability is particularly important. Sensor nodes are distributed linearly when monitoring linear structures, such as railway bridges, forming what is known as a Linear Wireless Sensor Network (LWSN). Although existing evaluation methods, such as enumeration and Binary Decision Diagram (BDD)-based methods, can be used to assess the reliability of various types of networks, their efficiency is relatively low. Therefore, we classified network states based on the number of failed nodes located at the network’s ends and analyzed the arrangement characteristics of nodes under different network states. This paper proposed a new reliability assessment method for LWSNs. This method is based on the combinatorial patterns of nodes and uses the concept of integer partitions to calculate the total number of states at different performance levels, applying probability formulas to assess network reliability. Compared to Multi-Valued Decision Diagram (MDD)-based evaluation algorithms, this method is suitable for large-scale LWSNs and offers lower time complexity.

## 1. Introduction

WSNs consist of multiple miniature sensors deployed within a designated area. These sensors can collect environmental data, including temperature, humidity, and pressure, and transmit this information wirelessly to other sensors or the base station [1,2]. Nowadays, wireless sensor networks have been widely used in various fields [3,4,5,6].

Wireless sensor networks (WSNs) have been widely applied with the development of wireless sensing technology. Linear wireless sensor networks (LWSNs) are characterized by sensor nodes distributed linearly, which are suitable for monitoring safety in linear areas such as bridges, pipelines, and rivers [7,8]. Ensuring the reliability of monitoring services is a primary research focus, where the network performance level is defined by the number of nodes capable of establishing direct or indirect connections with the base station. This paper proposes a novel reliability analysis method for LWSNs, analyzing the probabilities of LWSNs under different performance levels. Unlike existing enumeration and Binary Decision Diagram (BDD)-based analysis methods, this approach involves mathematically relating the states of the network, categorizing and arranging nodes to derive mathematical formulas for reliability analysis. The formulated mathematical expressions enable reliability analysis of larger-scale LWSNs with lower time complexity. Finally, the feasibility and efficiency of the new method are validated by comparing it with an MDD-based approach.

**Figure 1:**
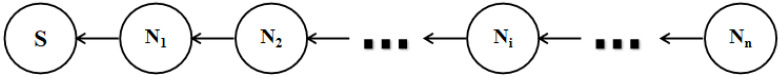
Thin LWSN.

LWSNs are a particular type of WSNs with a unique linear structure. As shown in Figure 1, a simple thin LWSN is presented. From the figure, it can be seen that the data transmission paths are minimal, so the survival status of nodes significantly affects the reliability of the network. When specific consecutive nodes in the network fail simultaneously, it forms voids, resulting in partial data being unable to be forwarded to the base station [9,10]. In [11], an efficient cluster head election scheme is proposed, considering initial energy, residual energy, and the optimal value of cluster heads for the election of the next group of cluster heads, significantly improving the network’s lifetime and residual energy. In [12], by defining special packet headers to update energy information and designing a re-clustering mechanism, the energy efficiency of the network is significantly improved.

The reliability of networks is crucial for the security status of monitoring facilities, and researchers have conducted extensive studies on this issue. The minimum cut method is often used to evaluate the reliability of random flow models [13]. In [14], a method for searching for the minimum cut in improved networks by adding new nodes between original network nodes was proposed, simplifying the search process. Unlike the minimum cut method, the state enumeration method is non-cut-set-based. It is the simplest among all network reliability evaluation algorithms [15], with high accuracy and efficiency. Additionally, the method based on binary decision diagrams (BDD) is a feasible evaluation algorithm, where BDD represents Boolean functions in a structured data form [16]. In [17], a method based on multi-valued decision diagrams (MDD) was proposed for analyzing multi-state systems, thus opening up a new path for the reliability evaluation of LWSNs. Mo et al. [18] expanded the MDD model and used it to analyze LWSNs with hybrid structures. Although MDD-based network evaluation algorithms are suitable for various structures of LWSNs, they are only suitable for networks with few nodes. This paper proposes a mathematical method-based evaluation algorithm applicable to LWSNs with only ordinary nodes. This method allows for a more significant total number of network nodes. Through comparative experiments with MDD-based evaluation algorithms, it has been proven that this algorithm has lower time complexity in evaluating the reliability of non-hybrid structured LWSNs and is suitable for large-scale LWSNs.

## 2. Related Works

In [7], two novel reliability evaluation metrics are proposed, the Generalized Terminal Reliability and the Average Generalized Terminal Reliability, and computational methods are developed based on algebraic graph theory and Monte Carlo simulation. A three-stage comprehensive optimization method is designed from a reliability perspective, focusing on determining the minimum number of sensor nodes, selecting task durations, and optimizing transmission power. The results validate the effectiveness of the proposed approach in balancing energy efficiency and connectivity while enhancing task cycle reliability. However, the research primarily focuses on the design and development phase, with limited exploration of reliability issues in other lifecycle stages (the deployment phase). In particular, further investigation is needed into dynamic modeling of interference and stochastic factors in complex environments and real-time control methods to ensure acceptable reliability.

Ref. [19] proposed a hierarchical belief rule-based (BRB) method for reliability evaluation of wireless sensor networks (WSNs) which comprehensively considers WSN failures and security factors. The method utilizes expert knowledge to construct initial rules and optimizes model parameters using experimental data, effectively addressing complex and uncertain information. Simulations and real-world case studies were used to validate the method, demonstrating strong consistency between evaluation results and actual reliability values, making it suitable for WSN reliability assessment in complex environments. However, the method has certain limitations, such as rule explosion caused by an excessive number of antecedent attributes, which requires optimization of the model’s internal structure.

Ref. [20] proposes an optimized deployment method for Local Positioning Systems (LPS) under sensor failure conditions. The method eliminates ambiguity by maximizing the distance between two possible solutions in four-sensor Time Difference of Arrival (TDOA) positioning and optimizes sensor node placement under both normal and failure conditions using the Cramer Rao Lower Bound and genetic algorithms. This approach significantly improves positioning accuracy and convergence region size in sensor failure scenarios while maintaining less than a 5% performance degradation during nominal operation, demonstrating its applicability in complex indoor and outdoor environments. However, the method has some limitations, including high computational complexity in the optimization process, potential challenges in practical deployment due to node quantity and hardware constraints, and the need for further validation and improvement in dynamic scenarios and real-time adaptability.

In [18], a flexible multi-state performance evaluation method based on Multi-Valued Decision Diagrams (MDD) is proposed for linear wireless sensor networks (LWSN) with hybrid structures. By sharing isomorphic submodel structures, a compact MDD model is constructed, effectively reducing model complexity. Compared to traditional exhaustive enumeration methods, this approach is more efficient and achieves a systematic study of LWSN at specific performance levels for the first time, providing theoretical support and practical validation for optimizing backbone node allocation strategies. However, the method is limited by computational resources when dealing with large-scale LWSNs, making it unable to generate precise models and relying on truncated MDD models for approximate analysis.

In summary, current reliability evaluation algorithms for WSNs face challenges such as implementation difficulty, high time and space complexity, and limitations in scalability for large networks. To address these issues, this paper proposes a reliability evaluation algorithm for LWSNs based on mathematical formulas to meet the needs of large-scale network assessments. By classifying network states and performing reliability analysis, the number of network states under different classifications is obtained. The reliability of the network at different nodes and maximum hop counts is then evaluated using the derived probability formulas. This method effectively reduces the evaluation complexity while ensuring accuracy. Comparisons show that, under the same conditions, it achieves higher evaluation efficiency than MDD-based evaluation algorithms, and the maximum number of nodes that can be evaluated is significantly increased.

## 3. Problem Statement

When monitoring infrastructure such as railways, bridges, and pipelines, many sensor nodes are typically deployed linearly in the monitoring area. These nodes form a LWSN by working collaboratively. As shown in Figure 1, the simplified LWSN model consists of a sink node located at the far left and *n* ordinary nodes, with node identifiers of $N_{i}$, where 0≤i≤n. The research in this article is based on thin LWSN and limited to thin LWSN. We make the following assumptions:

1. All ordinary nodes are produced in the same batch and are identical except for their identifiers $N_{i}$.
2. All ordinary nodes can only be in one of two possible states: 1, representing operational, and 0, representing failed.
3. All ordinary nodes follow the same failure probability distribution function.
4. The nodes are stationary and spaced evenly.

**Figure 2:**
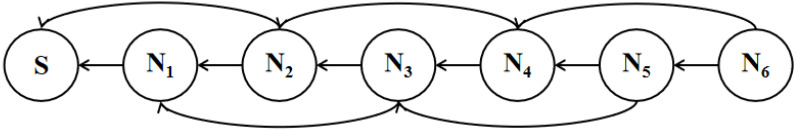
LWSN with *n* = 6 and *MJF* = 2.

During monitoring, there may be multiple failed nodes in the network. Define the network’s performance level as the total number of operational nodes in the current state denoted as *L*, where 0≤L≤n. Therefore, the network has a total of n+1 performance levels. Each performance level includes at least one network state, with each network state determined by the status of all nodes. The state vector $\vec{Z_{i}}$ represents the network state, where $\vec{Z}=\left(X_{1},X_{2},\cdots ,X_{i},\cdots ,X_{n}\right)$. $X_{i}$ is a Boolean variable representing node $N_{i}$’s status, with $X_{i}$ having a value of 0 or 1. Since each node has two possible states, there are $2^{n}$ different network states for an LWSN with n ordinary nodes. In practice, the communication range of nodes is limited, and the maximum number of hops covered by a node’s communication range is defined as the Maximum Jump Factor (MJF). The network will be interrupted when the number of consecutively failed nodes exceeds the *MJF*. Figure 2 shows a network with n=6 and *MJF*=2. Table 1 displays the network states included in each performance level.

Since the network has *MJF* = 2, the network will be interrupted when two consecutive nodes fail. Therefore, the states of the subsequent nodes will not impact the network and will be considered as failed. For $\vec{Z}=\left(X_{1},X_{2},\cdots ,X_{i},\cdots ,X_{n}\right)$, the probability of its occurrence is given by the following:

(1) $$P\left(Z\right)=\prod_{i=1}^{n}\left(X_{i}\left(1-f\left(t\right)\right)+\left(1-X_{i}\right)f\left(t\right)\right)$$

(2) $$f\left(t\right)=1-exp{\left(1-\lambda t\right)}^{\beta }$$

Here, f(t) represents the failure function of the node, i.e., Weibull failure time distributions. The time parameter *t* represents the total working time of the node during evaluation, while λ and β represent the physical parameters of the sensor. Due to different products or models, there may be different parameter values. In this article, it is assumed that all nodes are from the same batch and model of products, so the parameters are consistent. Therefore, according to the formula, the probability of the state (1,0,1,0,1,0) is $f^{3}{\left(1-f\right)}^{3}$. For the state (1,1,1,0,0,0), since the network will be interrupted after two consecutive nodes fail, the last failed node does not contribute to the probability calculation, so the probability for this state is $f^{3}{\left(1-f\right)}^{2}$.

The number of surviving nodes in a network directly affects its reliability. The performance level *L* of the network is the number of surviving nodes, so it is appropriate to use the probability of the network operating at different performance levels as an indicator for network reliability assessment. The reliability of the network at performance level *L* is given by the following equation:

(3) $$P^{\prime }\left(L\right)=\sum_{\forall Z}P\left(Z\right)$$

Since the total number of network states is $2^{n}$, the number of network states becomes extremely large when the number of nodes n is high, significantly reducing the evaluation algorithm’s efficiency. Therefore, this paper proposes an evaluation method that analyzes node arrangement patterns and uses integer partitioning to compute the total number of states at the current performance level. Combining this with probability formulas to assess network reliability effectively improves the evaluation efficiency.

## 4. Math-Based Evaluation Methods

### 4.1. Status Classification

Due to the complexity and variety of network states in large-scale LWSNs, we can divide the network states into *n* + 1 classes based on performance levels. For any given state, the number of nodes satisfies the following formula:

(4) $$n=L+F_{N}$$

**Figure 3:**
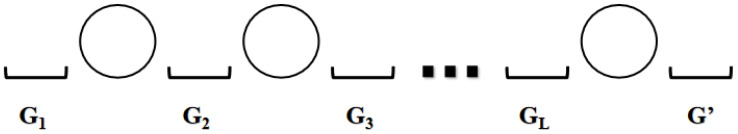
The gaps of LWSN.

Here, $F_{N}$ represents the total number of failed nodes. The number of operational nodes is also *L* for a network state with a performance level of *L*. In this case, consider the network state as placing the $F_{N}$ failed nodes into the L+1 gaps created after deploying the *L* operational nodes, as illustrated in Figure 3.

Due to the *MJF* constraint, after determining the *L* operational nodes, the number of failed nodes placed in each gap cannot exceed *MJF*, as this would lead to network disruption. However, this constraint applies only to the first *L* gaps. For the last gap, the number of failed nodes can exceed *MJF*, which will not affect the network’s performance level. We use $G_{j}$ to represent the number of failed nodes in the *j*-th gap, 1≤j≤L, and $G^{\prime }$ represents the number of failed nodes in the last gap. Since $G^{\prime }$ differs from $G_{j}$, we further classify the network states based on $G^{\prime }$ within the same performance level.

Let the network’s *MJF* be *m*; then, $G_{j}\in \left[0,m\right)$, and $G^{\prime }$ satisfies the following formula:

(5) $$G^{\prime }=F_{N}-\sum_{j=1}^{L}G_{j}=n-L-\sum_{j=1}^{L}G_{j}$$

Therefore, before classification, we need to determine the range of $G^{\prime }$, and then classify the network states based on different $G^{\prime }$ values. Since $G_{j}\in \left[0,m\right)$, $G^{\prime }$ reaches its minimum when m−1 failed nodes are placed at the first *L* vacant positions. In this case, $\sum_{j=1}^{L}G_{j}=\left(m-1\right)L$ and $G^{\prime }=F_{N}-\sum_{j=1}^{L}G_{j}=n-mL$. Of course, the obtained $\sum_{j=1}^{L}G_{j}$ in this case may exceed $F_{N}$, leading to $G^{\prime }<0$; however, in practice, $\sum_{j=1}^{L}G_{j}$ should be less than or equal to $\sum_{j=1}^{L}G_{j}$. Therefore, it needs to be set to $F_{N}$, resulting in $G^{\prime }=0$. The minimum value of $G^{\prime }$ is given as follows:

(6) $$G_{min}^{\prime }=max\left(0,n-mL\right)$$

Similarly, when all $G_{j}$ are set to 0, $G^{\prime }$ reaches its maximum value, which is $F_{N}$, as shown in the following:

(7) $$G_{max}^{\prime }=n-L$$

We can further classify the networks by considering different values of $G^{\prime }$. For example, in a network with n=6 and m=3, $G^{\prime }\in \left(0,3\right)$ when L=3; Table 2 provides the network states classified based on $G^{\prime }$.

### 4.2. State Analysis

According to the classification results in Table 2, each $G^{\prime }$ corresponds to at least one set of $G_{j}$, and each set of $G_{j}$ corresponds to a specific network state. When $G^{\prime }=0$, it indicates no failed nodes at the network’s end, meaning the network ends with operational nodes. When $\left(G_{1},G_{2},G_{3}\right)=\left(2,1,0\right)$, it means the number of failed nodes in the first three gaps is 2, 1, and 0, respectively, so the corresponding network state is (0,0,1,0,1,1). It is evident that when $G^{\prime }=0$, $\left(G_{1},G_{2},G_{3}\right)$ can be various permutations such as (0,1,2) or (1,1,1); this is also the case for $G^{\prime }=1,2,3,4$. Thus, $\left(G_{1},G_{2},G_{3}\right)$ represents different permutations of the partitions of $\left(n-L-G^{\prime }\right)$.

**Figure 4:**
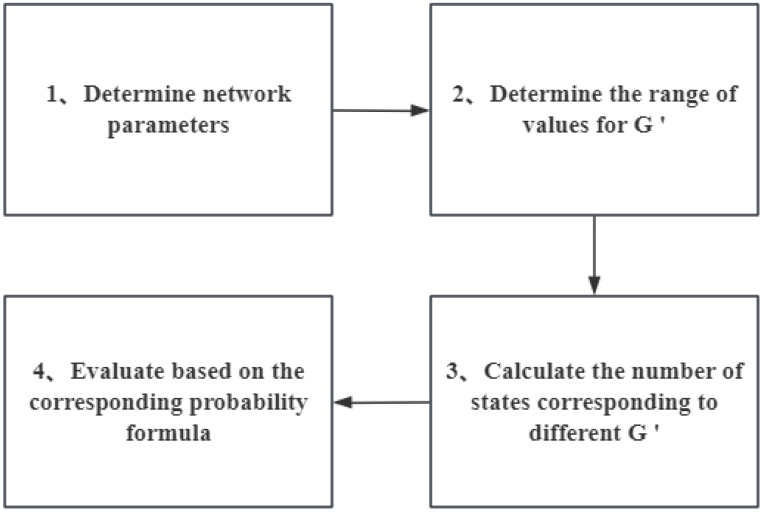
The workflow of evaluation.

Section 3 mentions that after *m* consecutive nodes fail, the states of subsequent nodes are no longer considered in the probability calculation. Therefore, for the current performance level, the relationship between $G^{\prime }$ and *m* determines the method for calculating network reliability (which will be detailed in Section 4.3). Therefore, the method proposed in this paper requires calculating the total number of states for different $G^{\prime }$ values separately and then using the corresponding probability formulas for reliability assessment. Figure 4 illustrates the general process of the proposed method.

### 4.3. Calculate the Number of States

In the previous section, we determined the range of $G^{\prime }$ and classified the network states according to $G^{\prime }$. Through state analysis, we found that each class of states corresponds to a set of $G_{j}$. Therefore, the total number of states can be obtained by calculating the permutations of $G_{j}$. Since $\sum_{j=1}^{L}G_{j}=FN-G^{\prime }$, we first need to obtain all possible $G_{j}$ combinations through integer partitioning. Let the sum of $G_{j}$ be *G* satisfied as follows:

(8) $$G=\sum_{j=1}^{L}G_{j}=n-L-G^{\prime }$$

Since $G_{j}\in \left[0,m\right)$, the numbers resulting from the partitioning of *G* should also fall within this range. Algorithm 1 outlines the implementation process for integer partitioning, where fun(x,y) represents the combinations of partitioning *x* with the maximum element not exceeding *y*. The main time cost of this evaluation method is consumed by the algorithm. In the average case, as seen from line 13 of the algorithm, where fun(x−y,y) and fun(x,y−1) are called, the total number of nodes *n* and the value of *MJF* decrease with each recursion. Specifically, *n* decreases by MJF in each iteration, and in each sub-recursion, *MJF* is reduced by 1. Therefore, the time complexity of the algorithm is approximately $O\left(2^{\frac{n}{m}+\frac{n}{m-1}+\frac{n}{m-2}+...+n}\right)\approx O\left(2^{nlnm}\right)$.
**Algorithm 1:** *y* splitting of *x*
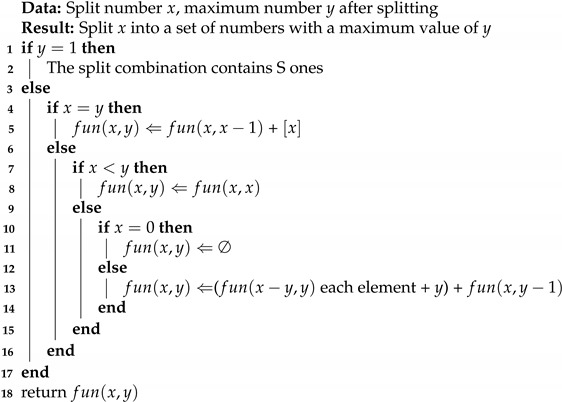


After partitioning, we need to pad some combinations with fewer elements than the number of intervals with zeros. For example, when calculating fun(3,2), the results are (1,2) and (1,1,1), but the required number of intervals is L=3. Therefore, for the combination (1,2), we need to pad it to (0,1,2) to meet the interval requirement.

After obtaining the combinations of $G_{j}$ through fun(G,m−1), we need to calculate the corresponding number of permutations, i.e., the different ways to place each $G_{j}$ into *L* intervals. The method for calculating the number of permutations is as follows:

(9) $$Sn=\frac{L!}{num_{0}!\cdot num_{1}!\cdot \ldots num_{m-1}!}$$

where Sn represents the number of permutations of $G_{j}$, or the number of states, and $num_{i}$ indicates the number of occurrences of *i* in the combination $\left(G_{1},G_{2},...,G_{L}\right)$, 0≤i≤(m−1). In Table 2, the six states corresponding to $G^{\prime }=1$ represent different permutations of two combinations: (0,0,2) and (0,1,1). For example, in the combination (0,0,2), the number of 0 is 2, 1 is 0, and 2 is 1. Therefore, ${num}_{0}=2$, ${num}_{1}=0$, and ${num}_{2}=1$. Substituting these values into Equation (9) gives the number of permutations for the combination (0,0,2), which is 3. Similarly, using the same method, the combination (0,1,1) also corresponds to 3 states. Using this formula, we can calculate the number of network states corresponding to each category of $G^{\prime }$, as shown in Table 3.

### 4.4. Probability Calculation

The execution probability of network states is related to the values of $G^{\prime }$ and *m*. For states where $G^{\prime }<m$, the state of each node is involved in the probability calculation. For example, in this case, P(1,0,1,0,1,0) = ${\left(1-f\right)}^{3}f^{3}$. When $G^{\prime }\geq m$, only the first *m* failed nodes among the $G^{\prime }$ nodes are involved in the calculation. The new formula for calculating the state probability is derived as follows.

(10) $$\begin{array}{c} P_{Z}=\left\{\begin{array}{cc} {\left(1-f\right)}^{L}\cdot f^{F_{N}}, & G^{\prime }\leq m\\ {\left(1-f\right)}^{L}\cdot f^{F_{N}-G^{\prime }+m}, & G^{\prime }>m \end{array}\right. \end{array}$$

The network’s reliability at the current performance level is obtained by calculating the probability for each type of network state using the probability formula multiplied by the corresponding number of states before summing. This method categorizes network states, significantly reducing the computational effort, and does not require obtaining specific network states during the evaluation process, thereby effectively improving evaluation efficiency.

## 5. Test and Analysis of the Results

In this section, a series of experiments are carried out to compare the existing reliability assessment algorithm based on MDD with the novel method proposed in [18], thereby validating the feasibility and effectiveness of the new approach. During the experiments, the time parameters t are varied at 1000, 5000, and 10,000 h, while the sensor parameters are set to $\lambda =3\times 10^{-5}$ and β=1.2. Initially, both algorithms are employed to evaluate the reliability of identical LWSNs, confirming the viability of the new algorithm. Subsequently, the efficacy of the new algorithm is substantiated through a comparative analysis of the evaluation efficiency of the two algorithms. Finally, a comparative evaluation of the two methods is conducted concerning their applicability.

### 5.1. Feasibility Verification

Due to the fact that the failure probability of a node satisfies Formula (2), it is related to the working hours and factory parameters of the node. Assuming that all nodes have the same factory parameters, for n=10 and MJF=2, we tested the reliability of the network at working hours of 1000, 5000, and 10,000 h, as shown in Table 4. The results are consistent with the evaluation results based on MDD algorithm, verifying the feasibility of this algorithm.

### 5.2. Efficiency Verification

**Figure 5:**
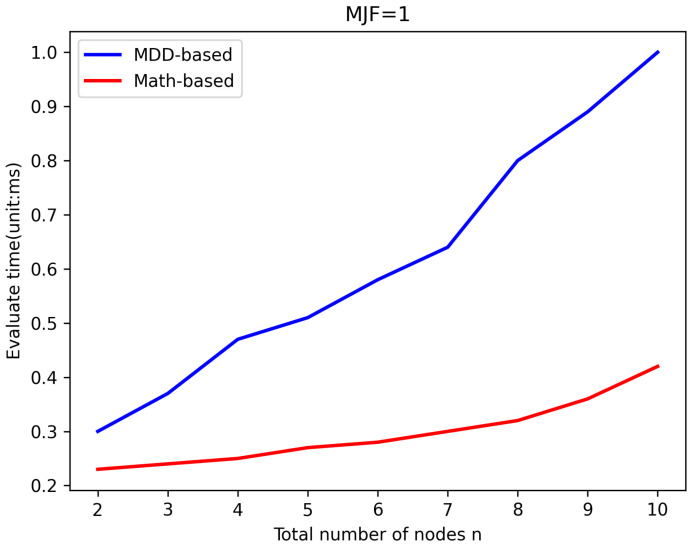
Efficiency comparison with *MJF* = 1.

**Figure 6:**
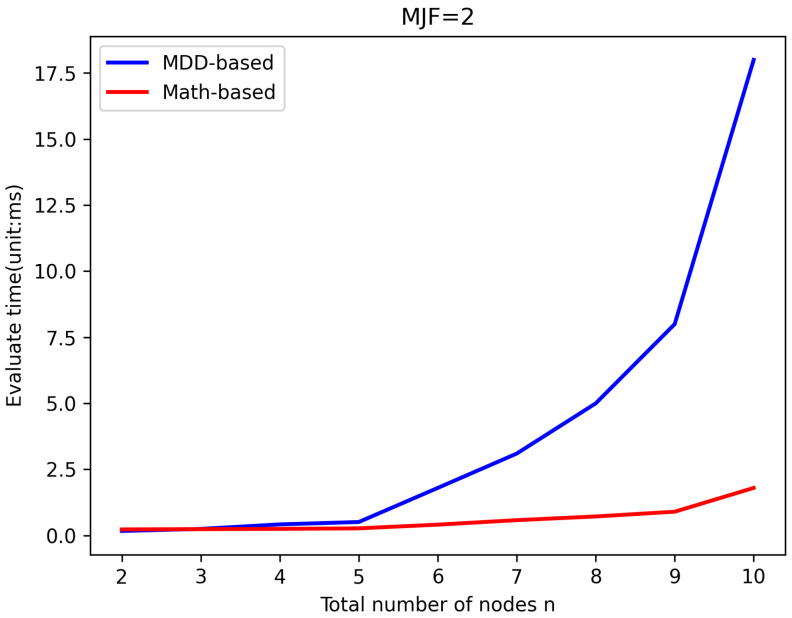
Efficiency comparison with *MJF* = 2.

**Figure 7:**
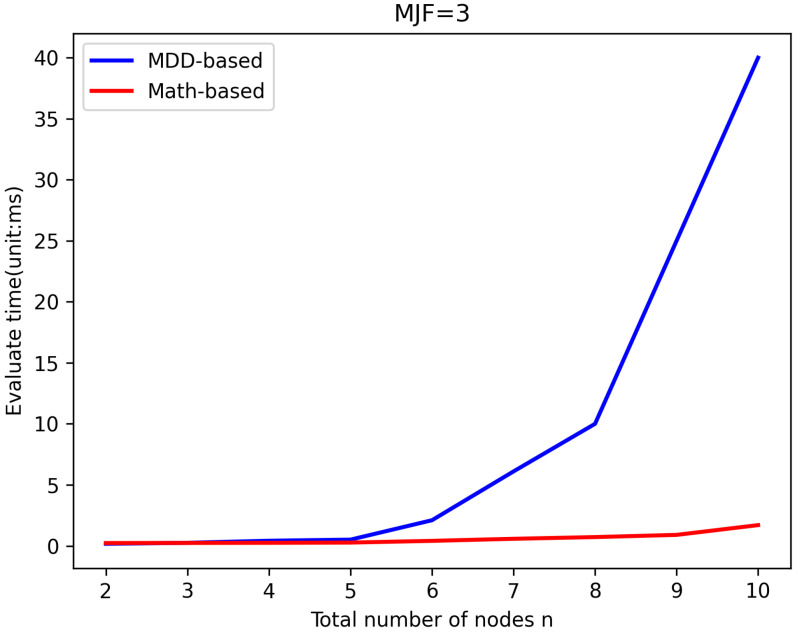
Efficiency comparison with *MJF* = 3.

**Figure 8:**
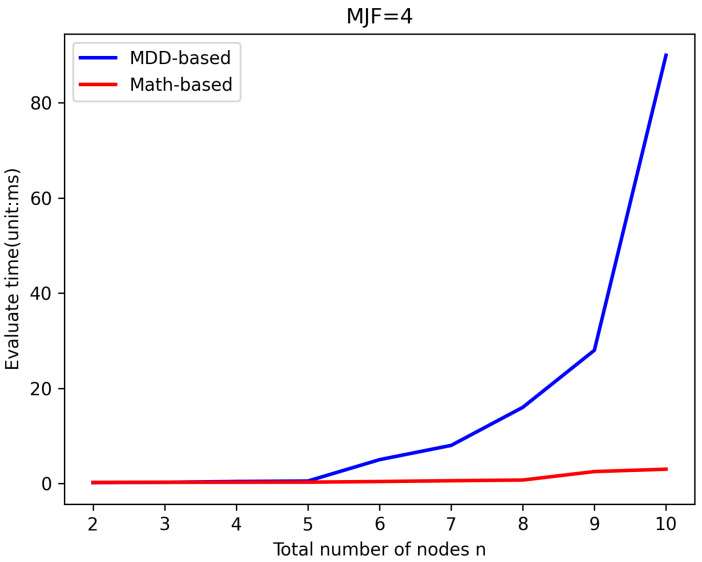
Efficiency comparison with *MJF* = 4.

LWSNs are extensively utilized to monitor infrastructure with linear structures. To prevent data loss caused by long transmission distances, LWSNs typically employ smaller *MJF*s in their design, which helps maintain efficient data transmission rates without overly complex network structures. To validate the efficiency of the new algorithm, it was compared with the existing MDD-based algorithm in evaluating the same LWSNs. The efficiency of the algorithms was reflected by comparing the time taken for evaluation. Figure 5, Figure 6, Figure 7 and Figure 8 illustrate the comparison of evaluation times for LWSNs with varying numbers of nodes at *MJF* values ranging from 1 to 4. The graphs demonstrate that as the number of nodes increases, the evaluation times for both algorithms also increase. This is expected as the increasing complexity of the network with more nodes leads to longer evaluation times. However, the new algorithm is less affected by the number of nodes. This can be attributed to the fact that the new algorithm does not require the construction and traversal of MDD models like the original algorithm. Therefore, in practical scenarios, the new algorithm better meets the demand for efficient evaluations.

### 5.3. Analysis of Applicable Scope

The efficiency of the new algorithm is significantly superior to that of the original algorithm. However, in actual LWSNs, the number of nodes varies. To better meet the requirements of most LWSNs, more extensive networks with a more significant number of nodes should be accommodated. We compared the two algorithms based on the maximum number of nodes that could be evaluated within a specified maximum evaluation time of 60 s. It was deemed unfeasible if the evaluation time was less than 60 s.

Table 5 and Table 6 reveal the range of nodes for valuable networks at *MJF* values of 2 and 3, respectively, for both algorithms. The new algorithm exhibits a broader applicability compared to the existing algorithm. For *MJF* = 2, the new algorithm can evaluate networks with a maximum of approximately 1000 nodes. In contrast, the original MDD-based algorithm is limited to networks with a maximum of up to 30 nodes, indicating that the efficiency of the new algorithm is less affected by the number of nodes. In contrast, for *MJF* = 3, the efficiency of both algorithms decreases. However, the new algorithm remains more efficient, and its performance decreases significantly, allowing for the evaluation of networks with a maximum of fewer than 60 nodes.

**Figure 9:**
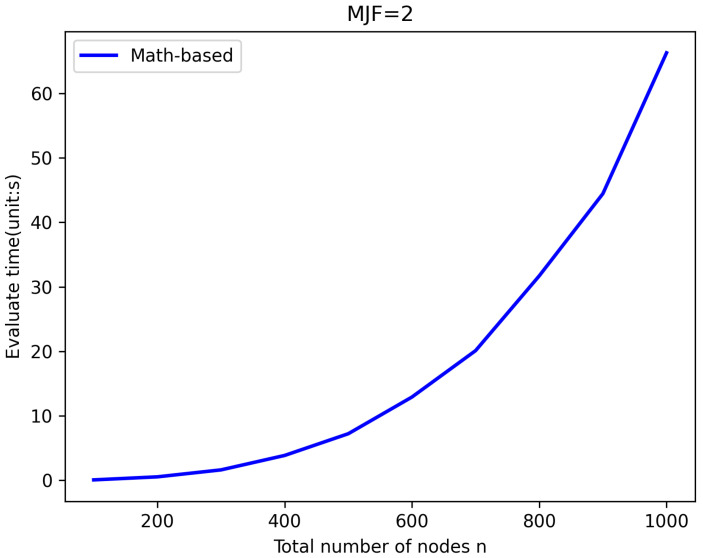
The maximum evaluation range when *MJF* = 2.

In practical scenarios, increasing MJF can significantly enhance the reliability of the network [21,22], but at the same time, the efficiency of network evaluation will be reduced due to the increase in network complexity. It is not difficult to find from the data in the Table 5 and Table 6 that in this method, the limit value of MJF is 2 when 50<n<1000 and is 3 when n<=50. Of course, this is also affected by the hardware foundation during the experiment. In Figure 9, the time efficiency of the evaluation using the math-based algorithm when MJF=2 is shown.

## 6. Conclusions

With the rapid development of sensing technology, LWSNs have gradually entered people’s vision. LWSNs play an essential role in monitoring infrastructure. However, research on the reliability of LWSNs is relatively scarce, and existing MDD-based evaluation algorithms achieve reliability evaluation by constructing a network-based MDD model. This method has shortcomings in efficiency and cannot pass the reliability evaluation of LWSNs with many nodes. Therefore, it has limitations in practical applications. This paper uses the unique linear structure of LWSNs to arrange and combine nodes in the network. Using the idea of integer splitting, the network’s reliability is evaluated through mathematical calculations, which is less affected by the number of nodes. Multiple experiments have proven that this method can evaluate LWSNs with a large number of nodes when *MJF* is within a generally small range. Therefore, it can meet the demand for reliability assessment in actual monitoring work. We plan to extend this method to more network models in the future, making its application scope more extensive. The *MJF* has a significant impact on the algorithm, limiting its applicability. In future work, we will further explore methods to mitigate the influence of *MJF* on efficiency to enable broader applications. Additionally, factors such as environmental interference and node mobility, which affect network reliability, will be considered to enhance the reliability of the evaluation results.

## Figures and Tables

**Table 1:** The network states.

L	Z (b = 0/1)
0	(0, 0, b, b, b, b)
1	(1, 0, 0, b, b, b), (0, 1, b, b, b, b)
2	(1, 1, 0, 0, b, b), (1, 0, 1, 0, 0, b), (0, 1, 1, 0, 0, b), (0, 1, 0, 1, 0, 0)
3	(1, 1, 1, 0, 0, b), (1, 1, 0, 1, 0, 0), (1, 0, 1, 1, 0, 0), (1, 0, 1, 0, 1, 0), (0, 1, 1, 1, 0, 0), (0, 1, 1, 0, 1, 0), (0, 1, 0, 1, 1, 0), (0, 1, 0, 1, 0, 1)
4	(1, 1, 1, 1, 0, 0), (1, 1, 1, 0, 1, 0), (1, 1, 0, 1, 1, 0), (1, 1, 0, 1, 0, 1), (1, 0, 1, 1, 1, 0), (1, 0, 1, 0, 1, 1), (0, 1, 1, 1, 1, 0), (0, 1, 1, 1, 0, 1), (0, 1, 1, 0, 1, 1), (0, 1, 0, 1, 1, 1)
5	(1, 1, 1, 1, 1, 0), (1, 1, 1, 1, 0, 1), (1, 1, 1, 0, 1, 1), (1, 1, 0, 1, 1, 1), (1, 0, 1, 1, 1, 1), (0, 1, 1, 1, 1, 1)
6	(1, 1, 1, 1, 1, 1)

**Table 2:** State classification at *L* = 3.

$G^{\prime }$	$\sum_{j=1}^{L}G_{j}$	$G_{1}$	$G_{2}$	$G_{3}$	Z
0	3	2	1	0	(0, 0, 1, 0, 1, 1)
		2	0	1	(0, 0, 1, 1, 0, 1)
		1	2	0	(0, 1, 0, 0, 1, 1)
		1	0	2	(0, 1, 1, 0, 0, 1)
		0	1	2	(1, 0, 1, 0, 0, 1)
		0	2	1	(1, 0, 0, 1, 0, 1)
		1	1	1	(0, 1, 0, 1, 0, 1)
1	2	2	0	0	(0, 0, 1, 1, 1, 1)
		0	2	0	(1, 0, 0, 1, 1, 0)
		0	0	2	(1, 1, 0, 0, 1, 0)
		1	1	0	(0, 1, 0, 1, 1, 0)
		1	0	1	(0, 1, 1, 0, 1, 0)
		0	1	1	(1, 0, 1, 0, 1, 0)
2	1	1	0	0	(0, 1, 1, 1, 0, 0)
		0	1	0	(1, 0, 1, 0, 0, 0)
		0	0	1	(1, 1, 0, 1, 0, 0)
3	0	0	0	0	(1, 1, 1, 0, 0, 0)

**Table 3:** The number of states.

$G^{\prime }$	G	$G_{j}$	${num}_{i}$	Sn
0	3	(0, 1,2)	(1, 1, 1)	6
		(1, 1, 1)	(0,3, 0)	1
1	2	(0, 0,2)	(2, 0, 1)	3
		(0, 1, 1)	(1,2, 0)	3
2	1	(0, 0, 1)	(2, 1, 0)	3
3	0	(0, 0, 0)	(3, 0, 0)	1

**Table 4:** Evaluation results for the reliability.

Performance Level	t = 1000 h	t = 5000 h	t = 10,000 h
L=0	2.180909 $\times \;10^{-4}$	9.515355 $\times \;10^{-3}$	4.412608 $\times \;10^{-2}$
L=1	2.180433 $\times \;10^{-4}$	9.424813 $\times \;10^{-3}$	4.217897 $\times \;10^{-2}$
L=2	2.179958 $\times \;10^{-4}$	9.335133 $\times \;10^{-3}$	4.031778 $\times \;10^{-2}$
L=3	2.179483 $\times \;10^{-4}$	9.246304 $\times \;10^{-3}$	3.853871 $\times \;10^{-2}$
L=4	2.179007 $\times \;10^{-4}$	9.158324 $\times \;10^{-3}$	3.683815 $\times \;10^{-2}$
L=5	2.178571 $\times \;10^{-4}$	9.100270 $\times \;10^{-3}$	3.582978 $\times \;10^{-2}$
L=6	2.193153 $\times \;10^{-4}$	1.059404 $\times \;10^{-2}$	4.788920 $\times \;10^{-2}$
L=7	3.793584 $\times \;10^{-4}$	3.314570 $\times \;10^{-2}$	1.205509 $\times \;10^{-1}$
L=8	7.163870 $\times \;10^{-3}$	1.548905 $\times \;10^{-1}$	2.475374 $\times \;10^{-1}$
L=9	1.291712 $\times \;10^{-1}$	3.872890 $\times \;10^{-1}$	2.515847 $\times \;10^{-1}$
L=10	8.617584 $\times \;10^{-1}$	3.583005 $\times \;10^{-1}$	9.460838 $\times \;10^{-2}$

**Table 5:** Comparison of evaluable ranges when *MJF* = 2.

*n*	11	15	20	25	30
MDD-Based (s)	0.01	0.18	2.71	38.15	—
*n*	200	400	600	800	1000
Math-Based (s)	0.52	3.84	12.91	31.7	66.23

**Table 6:** Comparison of evaluable ranges when *MJF* = 3.

*n*	11	15	20	21	25
MDD-Based (s)	0.06	0.91	25.53	49.82	—
*n*	20	30	40	50	60
Math-Based (s)	0.01	0.08	0.76	7.52	—

## Data Availability

Data are unavailable due to privacy constraints.
